# Intensive insulin treatment improves forearm blood flow in critically ill patients: a randomized parallel design clinical trial

**DOI:** 10.1186/cc8202

**Published:** 2009-12-09

**Authors:** Ivan Žuran, Pavel Poredoš, Rafael Skale, Gorazd Voga, Lucija Gabršček, Roman Parežnik

**Affiliations:** 1 Department of Angiology, Endocrinology and Rheumatology, General Hospital Celje, Oblakova ul. 5, 3000 Celje, Slovenia; 2 Clinical Department of Vascular Diseases, University Medical Centre, Ljubljana, Zaloška c. 2, 1000 Ljubljana, Slovenia; 3 Department of Intensive Internal Medicine, General Hospital Celje, Oblakova ul. 5, 3000 Celje, Slovenia

## Abstract

**Introduction:**

Intensive insulin treatment of critically ill patients was seen as a promising method of treatment, though recent studies showed that reducing the blood glucose level below 6 mmol/l had a detrimental outcome. The mechanisms of the effects of insulin in the critically ill are not completely understood. The purpose of the study was to test the hypothesis that intensive insulin treatment may influence forearm blood flow independently of global hemodynamic indicators.

**Methods:**

The study encompassed 29 patients of both sexes who were admitted to the intensive care unit due to sepsis and required artificial ventilation as the result of acute respiratory failure. 14 patients were randomly selected for intensive insulin treatment (Group 1; blood glucose concentration 4.4-6.1 mmol/l), and 15 were selected for conventional insulin treatment (Group 2; blood glucose level 7.0 mmol/l-11.0 mmol/l). At the start of the study (t_0_, beginning up to 48 hours after admittance and the commencement of artificial ventilation), at 2 hours (t_1_), 24 hours (t_2_), and 72 hours (t_3_) flow in the forearm was measured for 60 minutes using the strain-gauge plethysmography method. Student's t-test of independent samples was used for comparisons between the two groups, and Mann-Whitney's U-test where appropriate. Linear regression analysis and the Pearson correlation coefficient were used to determine the levels of correlation.

**Results:**

The difference in 60-minute forearm flow at the start of the study (t_0_) was not statistically significant between groups, while at t_2 _and t_3 _significantly higher values were recorded in Group 1 (t_2_; Group 1: 420.6 ± 188.8 ml/100 ml tissue; Group 2: 266.1 ± 122.2 ml/100 ml tissue (95% CI 30.9-278.0, *P* = 0.02); t_3_; Group 1: 369.9 ± 150.3 ml/100 ml tissue; Group 2: 272.6 ± 85.7 ml/100 ml tissue (95% CI 5.4-190.0, *P* = 0.04). At t_1 _a trend towards significantly higher values in Group 1 was noted (*P* = 0.05). The level of forearm flow was related to the amount of insulin infusion (r = 0.40).

**Conclusions:**

Compared to standard treatment, intensive insulin treatment of critically ill patients increases forearm flow. Flow increase was weakly related to the insulin dose, though not to blood glucose concentration.

**Trial Registration:**

Trial number: ISRCTN39026810.

## Introduction

Stress-induced hyperglycemia is a relatively common condition in patients admitted to intensive care units. It occurs in almost all patients with diabetes, as well as in patients with previously normal glucose metabolism [1]. Hyperglycemia (defined as a fasting plasma glucose >11.0 mmol/l) results from a reaction to a tissue injury or infection [2]. To date, the goal of hyperglycemia treatment has focused on maintaining glucose levels between 8.8 and 11.0 mmol/l. However, in a prospective, randomized, controlled study, it was shown that intensive insulin treatment maintaining glucose levels below 6.1 mmol/l significantly reduces both the mortality and the morbidity in critically ill patients in the surgical intensive care unit [3]. In another study of medical critically ill patients, morbidity but not mortality was reduced by intensive insulin treatment [4].

Recent data, however, indicate that intensive insulin therapy does not have a beneficial effect in critically ill patients and that it increases the risk of serious adverse events related to hypoglycemia; in the Normoglycemia in Intensive Care Evaluation-Survival Using Glucose Algorithm Regulation (NICE-SUGAR) study it was found that intensive glucose control increases mortality among patients treated in the intensive care unit [5,6]. Therefore, there is no definite answer of whether intensive glucose control has a long-term beneficial effect on the survival of critically ill patients, and the effect of insulin in these patients is not clearly understood. Most likely, insulin has different effects, and, among other factors, these effects are probably due to the improvement of vasodilation in peripheral circulation based on increased activity of the endothelial nitric oxide synthase (eNOS) [7].

The purpose of this study was to investigate if intensive insulin treatment in critically ill ventilated patients causes a change in forearm flow, and what is the relation between the forearm flow and the blood glucose concentration.

## Materials and methods

The study was conducted on patients admitted to the Department of Intensive Internal Medicine, General Hospital Celje, Slovenia, between January 2005 and December 2006. We included those critically ill patients who met the criteria for severe sepsis with acute respiratory failure requiring artificial ventilation. The following criteria for severe sepsis were considered: body temperature above 38°C or below 36°C, heart rate over 90 bpm, respiratory rate over 20 breaths/min or partial pressure of arterial carbon dioxide below 32 mmHg, leukocyte count over 12.0 × 10^9^/l or below 4.0 × 10^9^/l [8]. Those patients were included who met at least two criteria of sepsis. No diabetic patients were included in the study. Patients requiring artificial ventilation due to primary failure of respiratory muscles and those who required artificial ventilation due to brain injury were also excluded. Prior to inclusion, the patients' legal representatives signed written consent for participation in the study. The patients were randomized into two groups as regards the regulation of blood glucose: intensive (Group 1) and conventional (Group 2). In the conventional protocol the blood glucose concentration was maintained within the range of 7.0 to 11.0 mmol/l, while in the intensive protocol the concentration was maintained within the range of 4.4 to 6.1 mmol/l. The lower level (7 mmol/l) in patients receiving the conventional protocol was selected as per the proposal by the supervisory committee, because the recommendations at that time favoured blood glucose levels less than 8.3 mmol/l [9]. In a 50 ml syringe, 50 IU of human insulin for intravenous administration was diluted in a 0.9% solution of sodium chloride. The amount of infusion was adjusted according to the values of blood glucose concentrations in conformity with a previously published protocol [10]. The blood glucose concentration was determined hourly using the hexokinase method at the beginning of insulin treatment, and every two hours thereafter, except when the dose of insulin was adjusted; in this case, the next measurement was taken after one hour. The treatment was initiated within 48 hours of the start of artificial ventilation. Up to that point, the blood glucose concentrations were maintained in the 8.8 to 11.0 mmol/l range by means of subcutaneous administration of rapid-acting insulin or the infusion described above.

Hypoglycemia, a possible adverse event occurring during insulin treatment, was defined as a decrease in blood glucose concentration to values below 2.2 mmol/l, and was suspected in cases where the patient suffered sudden perspiration, convulsions, and change in heart rate or blood pressure. In these cases, administration of insulin was interrupted and blood was taken to determine the glucose levels. In addition, we performed a bedside test to determine the glucose values. If the blood glucose values were found to be below 2.2 mmol/l, we terminated the insulin infusion and the patient was intravenously administered 25 g of glucose in the form of a 50% solution.

All patients were continuously subjected to hemodynamic monitoring with the following measurements: continuous monitoring of the electrocardiography curve, and invasive measurement of arterial and central venous pressure. Cardiac output was continuously monitored by means of the thermodilution method (Edwards Lifesciences, Vigilance, Irvine, CA, USA).

Any additional monitoring was introduced by the principal physician, depending on the patient's clinical status.

The severity of the patients' clinical status was assessed by means of the Acute Physiology and Chronic Health Evaluation (APACHE) II score system routinely used for all patients treated at the department [11,12]. All patients were artificially ventilated with a Siemens Servo ventilator 300 set (Danvers, MA, USA) to pressure regulated volume control with a tidal volume of 5 to 7 ml/kg.

The patients were given food using a nasogastric tube as soon as possible, mostly after the initial 12-hour volume resuscitation. The food was administered between 6 a.m. and 10 p.m. During the overnight break in feeding the insulin dose was halved regardless of the insulin treatment protocol. Patients who did not tolerate enteral feeding received food in the form of a parenteral infusion of nutrients, and the insulin dose was adjusted based on the blood glucose levels.

The study was approved by the State Ethics Committee.

### Strain-gauge plethysmography

Measurements of forearm flow were performed by means of a plethysmograph (model EC5R, D.E. Hokanson, Inc., Bellevue, WA, USA). A detailed test procedure is described elsewhere [13,14]. Briefly, the patient was in a supine position with the upper body lifted by approximately 15°C. The forearm was positioned in the level of the right atrium (at 3/5 chest height). A 10 cm wide cuff was placed on the forearm and connected to the rapid cuff inflator. A mercury-filled clamp with a circumference 1.5 to 2 cm smaller than the forearm circumference was placed on the widest part of the forearm. A second 8 cm wide cuff was placed just above the wrist in order to block arterial inflow to the thermoregulatory area, in our case the hand. The upper arm cuff pressure was preset to 50 mmHg. After 10 seconds of inflation the cuff was deflated for five seconds. Prior to the measurement, the wrist cuff was inflated to the value 40 mmHg above the systolic pressure for the duration of a single measurement (approximately one minute). The plethysmographic curve was recorded and measurement was repeated every 10 minutes, with each individual measurement lasting one hour.

The instantaneous arterial flow was calculated manually by analysing the plethysmographic recording.

The values of the instantaneous arterial flow were expressed as ml/100 ml of tissue/min. To estimate the total forearm flow, the area under the 60-minute arterial flow curve was calculated. All arterial flow measurements were taken at the beginning of the study (t_0_), after 2 hours (t_1_), after 24 hours (t_2_), and after 72 hours (t_3_) between 8 a.m. and 9 a.m., with the exception of insulin infusion measurements, which were taken between 11 a.m. and 12 p.m.

### Laboratory tests

To determine blood glucose levels, blood was taken from an arterial catheter for hemodynamic monitoring every hour at the beginning of the study, and every two hours thereafter if the insulin infusion was not changed. Exceptionally, if hypoglycemia was suspected, a bedside test was performed to determine the glucose level from capillary blood; the test was always verified by collecting arterial blood. Serum glucose was determined on the Roche Modular (Hitachi Ltd, Tokyo, Japan) apparatus using the hexokinase method.

### Statistical model

The study was designed as a prospective, randomized, parallel study. Student's t-test of independent samples was used for comparisons between the two groups. Blood glucose concentrations showed a deviation from normal distribution; in this case, consequently, the comparisons between the groups were made using Mann-Whitney test. To compare categorical values, either the chi-squared test or Fisher's exact test was used, according to appropriateness. To calculate statistical differences in flows between the two groups of patients, the area under the flow curve during the one-hour measurement was considered as an individual piece of data. The area was calculated using the trapezoid rule [15].

The sample size was estimated at 30 patients based on findings from previously published data and on the basis of results from our own pilot study [16]. The data are expressed here as mean value ± standard deviation or, in the case of abnormal distribution, as the median, interquartile range or range between the minimum and maximum value. Linear regression analysis and the Pearson correlation coefficient were used to determine the levels of correlation. The value *P *< 0.05 was deemed as a statistically significant difference. Statistical calculations were carried out using the programme SPSS for Windows 10.0 (Chicago, Il, USA).

## Results

### Patient data

Twenty-nine patients were included in the study, 18 male and 11 female. 15 patients were randomly selected for conventional insulin treatment, and 14 were selected for intensive insulin treatment. The average age in the group of patients receiving intensive insulin treatment (Group 1) was 57.1 years (± 14.8), while in the group receiving conventional treatment (Group 2) the average age was 58.5 years (± 14.3) (*P *= 0.79). Group 1 consisted of 8 male and 6 female patients, and Group 2 consisted of 10 male and 5 female patients (*P *= 0.71). All the patients completed the study. In one patient, the arterial flow could not be measured after 24 hours because the patient could not be sufficiently sedated. One patient who had already been included in the study was excluded after 24 hours due to early completion of the treatment; this individual was replaced by another patient. Randomization was repeated for this patient. A comparison between the groups with respect to sex, age, initial serum glucose value and glycated haemoglobin (HbA_1_c) value and APACHE II shows that the groups did not differ according to these indicators (*P *= 0.70 and 0.48, respectively; Table 1).

**Table 1:** Demographic and physiological data of the two groups of patients

Variable	Group 1	Group 2	*P *value
Age (years)	57.1 ± 14.8	58.5 ± 14.3	0.79
Sex (male/female)	8/6	10/5	0.71
BMI (kg/m^2^)	31.1 ± 5.6	29.3 ± 3.7	0.31
HbA1c (%)	6.4 ± 0.7	6.5 ± 1.1	0.70
APACHE II (score)	21.4 ± 5.8	23.2 ± 5.6	0.48

The data are presented as mean values ± standard deviation.

APACHE = Acute Physiology and Chronic Health Evaluation; BMI = body mass index; HbA1C = glycated haemoglobin level.

The reasons and leading diagnoses for the patients' hospitalisation in Group 1 were: pneumonia in six patients, septic shock in four patients, and meningococcal meningitis in one patient. In Group 2, nine patients suffered from pneumonia, four from septic shock, and two from acute pancreatitis.

### Hemodynamic data at the time of admission and after 12-hour volume resuscitation

Immediately after admission the patients underwent volume resuscitation. Table 2 indicates the predominant use of a crystalloid infusion, that is a 0.9% solution of sodium chloride, which is a standard type of crystalloid used in our institution. The patients were also administered hydroxyethyl starch, although in significantly smaller doses. As the table shows, there was no difference between the two groups with respect to output data, type of volume treatment or hemodynamic response after 12 hours (Table 2).

**Table 2:** Hemodynamic parameters at the time of admission and after 12 hours of volume resuscitation, type of volume resuscitation in the initial 12 hours

Time	Variable	Group 1	Group 2	*P *value
Admission to intensive care unit	Heart rate (beat/min)	128.3 ± 19.4	118.0 ± 22.6	0.29
	Mean arterial pressure (mmHg)	65.9 ± 20.9	64.8 ± 21.4	0.91
	Central venous pressure (mmHg)	13.0 ± 5.7	12.1 ± 2.7	0.68
	Lactate (mmol/l)	3.6 ± 1.9	5.3 ± 3.2	0.18

Postresuscitation (12 hours)	Heart rate (beat/min)	105.0 ± 19.7	97.5 ± 16.9	0.28
	Mean arterial pressure (mmHg)	85.4 ± 15.9	84.6 ± 12.1	0.90
	Central venous pressure (mmHg)	16.1 ± 4.5	16.3 ± 5.7	0.92
	Lactate (mmol/l)*	2.6 ± 1.8	1.8 ± 0.7	0.18
	Cristalloid infusion (ml/kg/12 hours)	47.3 ± 25.6	49.6 ± 28.7	0.85
	Hydroxyethyl starch			
	Number of patients treated	3	5	0.23
	Cumulative dosage (12 hours)	11.5 ± 1.0	13.3 ± 2.1	0.18

Group 1 received intensive treatment and Group 2 received conventional treatment. Values are expressed as mean value ± standard deviation.

* value of lactate is measured 24 hrs after admission to intensive care unit.

### Hemodynamic monitoring of patients during the insulin treatment protocol

Table 3 shows key hemodynamic data and lactate values during the study. As is evident from the table, there were no statistically significant differences between the two groups.

**Table 3:** Hemodynamic data on patients at the beginning (t_0_), after 2 hours (t_1_), after 24 hours (t_2_)and after 72 hours (t_3_) in both groups of patients

Time	Variable	Group 1	Group 2	*P *value
t_0_	Heart rate (beat/min)	109.0 ± 20.5	95.8 ± 20.7	0.16
	Mean arterial pressure (mmHg)	87.9 ± 16.1	90.2 ± 12.0	0.66
	Central venous pressure (mmHg)	16.6 ± 3.7	15.8 ± 4.2	0.58
	Cardiac index (l/min/m^2^)	4.4 ± 1.4	3.9 ± 1.7	0.55
	Lactate (mmol/l)	2.6 ± 1.8	1.8 ± 0.7	0.17

t_1_	Heart rate (beat/min)	107.0 ± 20.6	93.3 ± 20.4	0.15
	Mean arterial pressure (mmHg)	92.0 ± 19.6	88.7 ± 14.2	0.69
	Central venous pressure (mmHg)	17.0 ± 4.6	16.2 ± 3.5	0.68
	Cardiac index (l/min/m^2^)	4.3 ± 1.6	3.7 ± 0.5	0.39

t_2_	Heart rate (beat/min)	98.5 ± 17.5	93.3 ± 15.7	0.41
	Mean arterial pressure (mmHg)	85.4 ± 15.9	91.5 ± 12.2	0.25
	Central venous pressure (mmHg)	16.9 ± 4.0	14.6 ± 5.4	0.20
	Cardiac index (l/min/m^2^)	4.4 ± 1.3	3.9 ± 1.5	0.46
	Lactate (mmol/l)	1.8 ± 1.1	1.2 ± 0.5	0.09

t_3_	Heart rate (beat/min)	97.1 ± 18.2	96.7 ± 19.5	0.95
	Mean arterial pressure (mmHg)	95.1 19.5	95.0 ± 19.6	0.99
	Central venous pressure (mmHg)	13.3 ± 5.3	12.1 ± 4.5	0.49
	Cardiac index (l/min/m^2^)	4.6 ± 1.4	3.9 ± 1.0	0.25
	Lactate (mmol/l)	1.6 ± 1.1	1.0 ± 0.4	0.19

Group 1 received intensive treatment and Group 2 received conventional treatment. Vales are expressed as mean ± standard deviation.

### Comparison of both groups with respect to therapeutic procedures

Table 4 shows a comparison of all therapeutic procedures throughout the duration of the treatment. As is evident, the only difference between the groups was the total daily dose of insulin at t_2_. The difference remained significant at t_3 _(*P *< 0.01).

**Table 4:** Comparison of Groups 1 and 2 (intensive vs. conventional protocol) with respect to key therapeutic procedures

Type of treatment	Group 1	Group 2	*P*-value
**Ventilatory support**			
FiO_2_	0.5 ± 0.2	0.4 ± 0.1	0.25
PEEP(cmH_2_O)	7.5 ± 1.5	9.0 ± 3.6	0.54
**Nutritional support**			
Total daily caloric intake (kcal/kg/day)	19.5 ± 3.4	23.7 ± 8.1	0.14
Carbohydrate caloric intake (kcal/kg/day)	9.7 ± 4.1	10.9 ± 4.9	0.56
Enteral nutrition			
Number of patients (%)	9 (64)	10 (66)	0.70
Enterak daily caloric intake (kcal/kg/day)	11.3 ± 7.4	17.3 ± 6.4	0.11
**Hemodynamic support (norepinephrine infusion)**			
Number of patients treated	8	5	0.27
norepinephrine infusion rate (μg/kg/min)	0.3 ± 0.2	0.4 ± 0.2	0.43
Antibiotics*			
Number of patients treated	14	15	1.0
**Insulin infusion (U/day)**	140.7 ± 56.3	67.2 ± 44.9	< 0.01
**Corticosteroid treatment (methylprednisolone)**			
Number of patients treated	10	7	0.47
methylprednisolone dosage (mg/day)	160 ± 84	200 ± 0	0.27
**Hemodialysis**			
Number of patients	2	1	0.60
**Blood transfusion**			
Number of patients	1	1	0.62

The numerical data refers to the period 24 hours following the start of insulin treatment. The number of patients receiving antibiotics, hemodialysis and blood transfusion applies to the entire duration of the treatment.

FiO2 = fraction of inspired oxygen; PEEP = positive end-expiratory pressure

* treatment with at least one antibiotic is taken into account

### Glucose concentration at different check-ups

Table 5 shows the serum glucose values as well as insulin dose in Group 1 and Group 2 at individual measurements.

**Table 5:** Serum glucose concentrations at the beginning (t_0_), after 2 hours (t_1_), after 24 hours (t_2_), and after 72 hours (t_3_) in both groups of patients and simultaneous insulin doses expressed in U/h

Time	Variable	Group 1	Group 2	*P*-value
t_0_	Serum glucose level (mmol/l)	9.4 (6.7-13.6)	8.8 (4.6-21.0)	0.03
	Insulin infusion (U/h)	4.0 ± 3.2	3.8 ± 3.2	0.89
t_1_	Serum glucose level (mmol/l)	6.8 (4.3-9.4)	8.3 (4.3-19.4)	0.01
	Insulin infusion (U/h)	9.7 ± 3.6	3.6 ± 2.7	< 0.01
t_2_	Serum glucose level (mmol/l)	6.0 (3.9-8.7)	7.9 (5.5-12.1)	< 0.01
	Insulin infusion (U/h)	6.2 ± 3.3	3.4 ± 3.4	0.03
t_3_	Serum glucose level (mmol/l)	5.2 (3.7-11.4)	7.6 (4.6-11.3)	< 0.01
	Insulin infusion (U/h)	4.9 ± 3.7	2.2 ± 2.7	0.03

The data are shown as medians and interquartile ranges or mean ± standard deviation.

At the beginning of the study, serum glucose concentrations were lower in Group 2 (*P *= 0.03). At t_1_, significantly lower levels were recorded in Group 1 as compared with those in Group 2 (*P *= 0.01). The difference in concentrations remains significantly higher in the intensively treated group at t_2 _and t_3 _(*P *< 0.01).

For the duration of the study (72 hours) no clinical or laboratory signs of hypoglycemia were recorded. The lowest measured level of serum glucose was 3.8 mmol/l (in a Group 1 patient).

A comparison of insulin doses at individual flow measurements indicates that at time t_0 _the doses were not statistically different (*P *= 0.89), while at times t_1_, t_2 _and t_3 _Group 1 patients were administered significantly larger doses (*P *< 0.01, 0.03, and 0.03, respectively).

### Total arterial flow in the forearm of investigated patients of both groups at different check-ups

The 60-minute forearm flow at the start of the trial (t_0_) did not differ between Group 1 and Group 2 (305.0 ± 137.8 ml/100 ml tissue vs. 255 ± 104.2 ml/100 ml tissue; *P *= 0.28).

**Figure 1:**
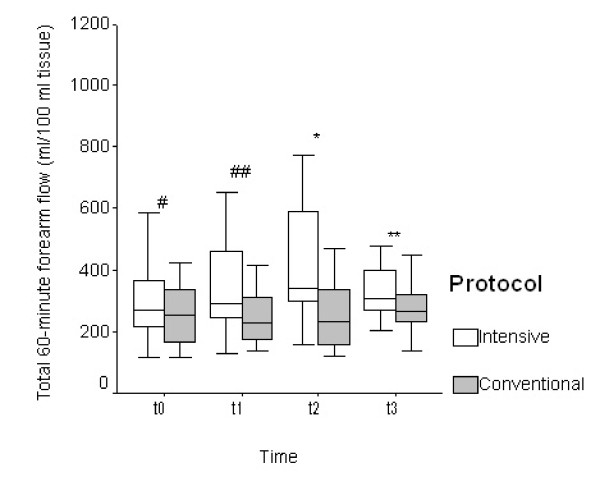
Total 60-minute blood flow in the forearm at the beginning (t_0_), after 2 hours (t_1_), after 24 hours (t_2_), and after 72 hours (t_3_) in both groups of patients. Values are shown as medians (horizontal bars inside the box) with the 25th and 75th percentile (upper and lower frame of the box) and the 5th and 95th percentile (bars). # *P *= 0.28, ## *P *= 0.05, * *P *= 0.02, ** *P *= 0.04.

Statistically significant higher values in the total 60-minute arterial flow were found at t_2 _and t_3_, while at t_1 _only a trend towards increased flow in the intensively treated group was indicated (Figure 1). At t_2_, the value of 60-minute arterial flow was 420.6 ± 188.8 ml/100 ml of tissue in Group 1 and 266.1 ± 122.2 ml/100 ml of tissue in Group 2 (95% confidence interval (CI) = 30.9 to 278.0; *P *= 0.02), and at t_3 _369.9 ± 150.3 ml/100 ml of tissue vs. 272.6 ± 85.7 ml/100 ml of tissue (95% CI = 5.4 to 190.0; *P *= 0.04).

At t_1 _a trend towards a significant higher flow in Group 1 was observed (Group 1: 367.1 ± 192.7 ml/100 ml of tissue; Group 2: 253.0 ± 90.6 ml/100 ml of tissue; 95% CI = 0.6 to 227.5; *P *= 0.05).

### Interrelationship between blood flow and rate of insulin infusion

In determining correlations between independent and dependent variables we made use of linear regression analysis and Pearson's correlation. Independent variables were defined as those that were found to influence the dependent variables such as the 60-minute flow and maximum instantaneous forearm flow in previous studies.

**Figure 2:**
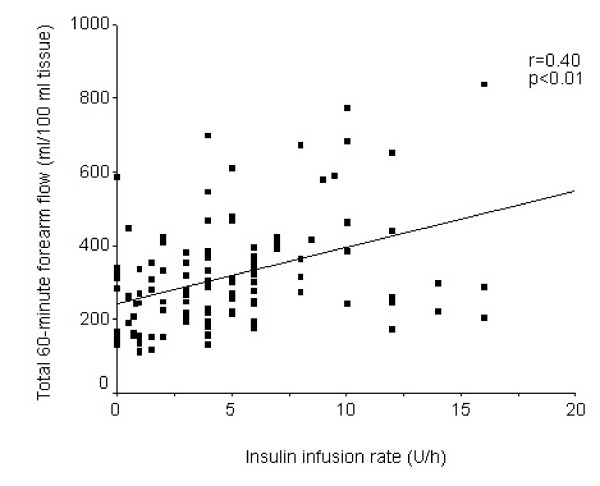
Interrelationship between the insulin infusion rate and the total 60-minute arterial flow.

Linear regression analysis confirmed the linear correlation between the rate of insulin infusion in U/h and the 60-minute arterial flow. Figure 2 shows that the flow increases in relation to the insulin infusion (r = 0.40, *P *< 0.01).

Conversely, no correlation was found between the glucose concentration and forearm flow (r = -0.054, *P *= 0.57).

## Discussion

To our knowledge, our study was the first to investigate the influence of intensive insulin treatment on forearm flow in critically ill, artificially ventilated patients. We found a significant flow increase 24 and 72 hours after the start of intensive treatment, whereas two hours after the start there was a borderline increase. With respect to the type of treatment, our groups differed only in the quantity of insulin administered to the patients within 24 hours. The initial volume resuscitation was carried out primarily with a crystalloid infusion, while a comparable number of patients also received low doses of hydroxyethyl starch. A recent study showed treatment with this colloid to be inappropriate due to increased incidence of adverse effects on the renal function and coagulation, increased need for blood transfusions, and adverse effects on survival [6]. During the study there were no differences in global hemodynamic parameters or in vasoactive norepinephrine treatment. During our study, no severe hypoglycemia was observed; this fact contrasts with the most recent studies, which have recorded 6.8% to 17% of severe hypoglycemic incidents [5,6]. The most probable explanation for the absence of hypoglycemia in our study is the relatively small sample of patients and the short period of intensive insulin treatment in comparison to other studies. The flow was in a linear, although weak, interrelationship with the rate of the insulin infusion. Conversely, our study did not confirm the interrelationship between the glucose concentration and forearm flow, which cannot be definitively explained. In another study the influence of intraarterial insulin infusion on protein synthesis in skeletal muscles in the legs was investigated in patients with burns [16]. In addition to the increased utilization of amino acids, they also found that the flow in the legs increased significantly.

The increase of blood flow could be related to the improvement of endothelial function.

The influence of insulin on the endothelial function has been studied extensively and it has been shown that the influence appears through the activation of eNOS [17-19]. The flow in skeletal muscles increases in two phases: first the dilatation of terminal arterioles triggers capillary recruitment within minutes, and in the second phase larger arteries dilate and the flow increases, the effect reaching its peak after two hours [20,21]. In sepsis, the stimulation of eNOS is inhibited and consequently the response of the endothelium on the insulin is limited [22]. In our study there was a nonsignificant increase in blood flow two hours after the start of the treatment. This could be a result of a delayed response, especially of large arteries, to the insulin infusion.

Our study indicates that insulin treatment improves skeletal muscle blood flow. The weak linear relation between the amount of the infused insulin and forearm flow in our study indicates that the regulation of the flow through skeletal muscles has been preserved and that it may be increased by means of therapeutic procedures such as insulin infusion. This presumption is in agreement with findings of Van den Berghe and colleagues, who showed that intensive insulin treatment significantly reduces the mortality and morbidity of critically ill patients [3,4]. However, recent data mitigate the positive effects of intensive insulin treatment of critically ill patients or suggest that a goal of normoglycemia does not necessarily benefit critically ill patients and may be harmful (the NICE-SUGAR study) [5]. These findings could mean that increased blood flow in the forearm is not an indicator of improvement of all perfusion (especially vital organs) in critically ill patients, but can be an indicator of the re-distribution of blood flow. The different findings in our study in comparison to the NICE-SUGAR study could also be a consequence of the duration of the follow-up period. In our study, we only followed patients for 72 hours. One possible explanation, thus, is that intensive glucose control has time-limited positive homodynamic effects (up to some days), and that afterward the positive effects of intensive insulin treatment are concealed by a higher complication rate related to adverse events, especially hypoglycemia.

There are some limitations to our study: the sample of patients was relatively small and the study was not completely blind; following randomization, the patients' principal physicians and nursing staff were informed of the type of insulin treatment protocol. During flow measurements, bias was minimised by coding the plethysmographic recordings and independent calculations of flow measurements.

## Conclusions

Compared with conventional treatment, the intensive treatment of critically ill patients with insulin results in increased arterial flow in the forearm. An increase in blood flow was indicated in the group of intensively treated patients after two hours, and became significantly greater after 24 hours and 72 hours. The increase of blood flow in the forearm is in a weak linear relationship to the rate of insulin infusion, although no relation with the glucose concentration was found. Based on our findings, it may be concluded that a certain increase in flow may be reached with insulin doses, which do not cause a dramatic reduction in blood glucose values below the acceptable level. Further research is required to determine long-term effects of the increase in blood flow in muscles during intensive insulin treatment of critically ill patients.

## Key messages

• Intensive insulin treatment of critically ill patients improves forearm flow.

• Changes in global hemodynamic indicators do not affect the increase in flow.

• The increase in flow is in a weak linear correlation with the insulin dose; however, there is no correlation between the flow increase and the concentration of glucose in the blood.

• The effect of insulin on the flow is short-term and is reduced within 72 hours from the start of intensive treatment.

• The clinical significance of hemodynamic effects of insulin will have to be evaluated.

## Abbreviations

APACHE: Acute Physiology and Chronic Health Evaluation; CI: confidence interval; eNOS: endothelial nitric oxide synthase; HbA_1_c: glycated haemoglobin.

## Competing interests

The authors declare that they have no competing interests.

## Authors' contributions

IŽ conceived the study, participated in the design of the study, coordinated the study implementation, drafted the manuscript, and participated in statistical analysis. PP participated in the design of the study, supervised the study, and helped to draft the manuscript. RS participated in the design of the study, performed randomization of the patients, and participated in implementation of the study. GV participated in the design of the study, and participated in the implementation of the study. LG and RP participated in implementation of the study.
